# The association between the triglyceride-glucose index and bone turnover markers in osteoporotic fractures patients aged 50 and above who are hospitalized for surgical intervention: a retrospective cross-sectional study

**DOI:** 10.3389/fendo.2024.1418271

**Published:** 2024-09-18

**Authors:** Jian Xu, Shao-han Guo, Min-zhe Xu, Chong Li, Ya-qin Gong, Ke Lu

**Affiliations:** ^1^ Department of Orthopedics, The First People’s Hospital of Kunshan, Gusu School, Nanjing Medical University, Suzhou, Jiangsu, China; ^2^ Department of Orthopedics, Affiliated Kunshan Hospital of Jiangsu University, Suzhou, Jiangsu, China; ^3^ Information Department, Affiliated Kunshan Hospital of Jiangsu University, Suzhou, Jiangsu, China

**Keywords:** P1NP, β-CTX, BTMs, osteoporotic fractures, TyG index

## Abstract

**Objective:**

To evaluate the correlation between the triglyceride-glucose (TyG) index and bone turnover markers (BTMs) in osteoporotic fractures (OPFs) patients hospitalized for surgical intervention.

**Methods:**

A retrospective cross-sectional study was conducted on 3558 OPFs patients hospitalized for surgical intervention between January 2017 and July 2022. The study obtained baseline values for various biomarkers and covariates, including fasting blood glucose, β-C-terminal telopeptide of type I collagen (β-CTX), procollagen type 1 N-terminal propeptide (P1NP), triglycerides, age, sex, body mass index, smoking, drinking, low-density lipoprotein, high-density lipoprotein, aspartate aminotransferase, uric acid, the score of American society of anesthesiologists, homocysteine, parathyroid hormone, apolipoprotein B, apolipoprotein A, magnesium, phosphorus and calcium. Multiple linear regression, curve fitting, threshold effects, and subgroup analyses were also applied.

**Results:**

After adjusting for covariates in the regression analysis, the results revealed a negative correlation between β-CTX and P1NP levels and the baseline TyG index. Specifically, a one-unit increase in the TyG index was associated with a reduction in β-CTX levels of -0.06 (95% CI: -0.10, -0.01; P-value = 0.012) and a reduction in P1NP levels of -4.70 (95% CI: -9.30, -0.09; P-value = 0.046). Additionally, the inflection points for the nonlinear correlation between the TyG index and β-CTX and P1NP were found to be K = 6.31 and K = 6.63, respectively.

**Conclusion:**

The study demonstrated a negative, non-linear relationship among the TyG index, β-CTX and P1NP in OPFs patients hospitalized for surgical intervention. These findings suggest that elevated TyG index levels may adversely affect bone turnover, potentially contributing to the progression of OP.

## Introduction

Osteoporosis (OP) is a prevalent metabolic bone disorder in the elderly, characterized by a gradual and continuous reduction in bone density and increased susceptibility to skeletal fragility (1). It is characterized by a bone mineral density (BMD) that is ≤ 2.5 standard deviations (SD) beneath the average peak of bone weight of normal adults (2, 3). Fragility fractures associated with OP are estimated to affect one-fifth of males and one-third of females aged 50 and above (4, 5). Currently, OP imposes an annual economic burden of $650 billion in the United States, Canada, and Europe, and this number is rapidly increasing (4, 6). Therefore, identifying and mitigating factors contributing to OP and improving overall bone health is crucial for public health and socioeconomic efforts.

Aging, obesity, and insulin resistance have been shown to cause pathogenic bone turnover and disruption of bone homeostasis (7, 8). The hyperinsulinemic-euglycemic clamp (HIEC) is usually recognized as the best method for assessing insulin resistance (IR); yet, its time-consuming and complex design limits its use in large clinical settings (9). To address these constraints, the triglyceride-glucose (TyG) index, which is measured as Ln [triglyceride (TG) (mg/dL) × fasting blood glucose (FBG) (mg/dL)/2] (7), has been recently recognized as a promising alternative biomarker for IR (10, 11). Recent studies have revealed a link between the TyG index and several conditions, including coronary artery disease (12), depression (13), hypertension (14), ischemic stroke (9), heart failure (15), liver fibrosis (16), kidney stone (17) and erectile dysfunction in men (18). Nevertheless, the correlation between this index and OP fractures (OPFs) patients hospitalized for surgical intervention remains unexplored.

Bone turnover markers (BTMs) are indicators of bone remodeling rates. They are byproducts of the bone remodeling process and can be measured in urine or serum (19). The International Osteoporosis Foundation (IOF) and the International Federation of Clinical Chemistry (IFCC) have identified serum β-C-terminal telopeptide of type I collagen (β-CTX) and procollagen type 1 N-terminal propeptide (P1NP) as reference indicators for bone development and resorption. These indicators are suggested for assessing fracture risk and tracking treatment for OP, as they can change with the progression of the disease (19). Both β-CTX and P1NP are degradation products of type I collagen. P1NP is released by osteoblasts during bone formation, while β-CTX is secreted by osteoclasts during bone resorption. As such, both markers are valuable for evaluating bone development and resorption (20).

Previous research has explored insulin resistance-related dysregulation of bone homeostasis in various populations, including patients with type 2 diabetes (7), non-diabetic Koreans (10), adult Americans (21), middle-aged and elderly non-diabetic Chinese individuals (22), and postmenopausal females (23). These studies have also investigated the relationship between the TyG index and bone mineral density (BMD). However, the connection between the TyG index and BTMs in OPFs patients hospitalized for surgical intervention has not been thoroughly investigated or supported by evidence. Therefore, the primary aim of the current study was to examine the potential correlation between the TyG index and BTMs.

## Materials and methods

### Ethical consideration

The study was carried out according to the Declaration of Helsinki and was approved by the Affiliated Kunshan Hospital of Jiangsu University (AKHJU) (2021-06-015-K01). During data analysis, patient information was kept confidential from the investigators. Before the study’s commencement, written consent was obtained from all participants.

### Research participants and design

**Figure 1:**
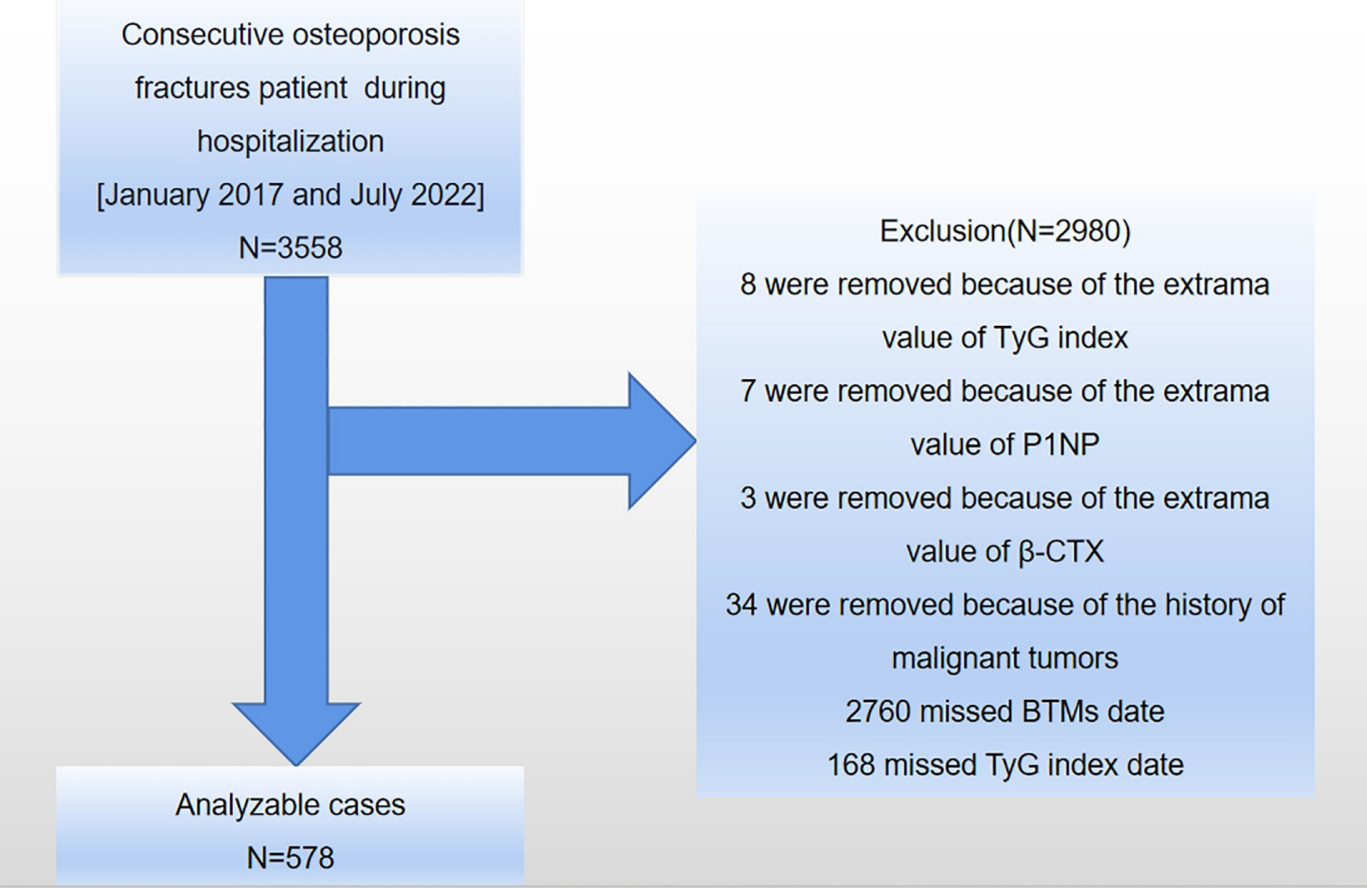
A diagrammatic representation of the study’s design. TyG index, triglyceride-glucose index; P1NP, type I procollagen N-terminal propeptide; β-CTX, type I collagen β-C-terminal telopeptide; BTMs, bone turnover markers.

This study was carried out at the AKHJU in Jiangsu Province, China. Electronic patient records were obtained for all individuals aged 50 and above who had recently been diagnosed with osteoporotic fractures (OPFs) requiring hospitalization between January 1, 2017, and July 27, 2022. An OPF was defined as a fragility fracture of the wrist, proximal humerus, hip, or vertebra that required hospitalization for surgical intervention and was diagnosed using the 10th revision of the International Statistical Classification of Diseases and Related Health Problems (ICD-10), specifically codes starting with S22, S32, S42, S52, or S72 (24). A total of 3,558 OPFs patients hospitalized for surgical intervention were included in the study. Exclusions were made for patients who: 1) did not provide data on the TyG index or BTMs, 2) had extreme values for TyG index or BTMs, 3) were under 50 years of age, 4) had a history of malignant tumors or psychiatric disorders, 5) had experienced myocardial infarction, cerebral hemorrhage, severe hepatic or renal insufficiency, acute infection, or stress within the past 3 months, or 6) were taking medications that could impact bone metabolism (7). A total of 578 patients met the inclusion criteria for the current analysis (refer to **Figure 1**).

### Study variables

The mentioned Ln [TG (mg/dL) × FBG (mg/dL)/2] (7) formula was used to determine the TyG index. A Beckman AU5800 automated biochemistry analyzer was employed to observe FBG using the hexokinase method and TG via the GPO-POD method. An electrochemiluminescence immunoassay was conducted on a Roche Cobas 8000 analyzer to quantify β-CTX and P1NP. The following covariates were included in the analysis: body mass index (BMI), sex, age, smoking, drinking, homocysteine, American Society of Anesthesiologists (ASA) score, aspartate aminotransferase (AST), uric acid (UA), magnesium (Mg), phosphorus (P), calcium (Ca), triglycerides (TG), fasting blood glucose (FBG), low-density lipoprotein (LDL), high-density lipoprotein (HDL), parathyroid hormone (PTH), apolipoprotein B (Apo B), and apolipoprotein A (Apo A). The Beckman AU5800 automated biochemical analyzer was used for further analysis. AST and homocysteine levels were measured using the enzymatic method, while UA was assessed with the uricase-peroxidase method. Ca was measured using the arsenazo III method, Mg with the dimethylaminoazobenzene method, and P using the phosphomolybdate method. HDL and LDL were measured using the direct method, and Apo A and Apo B were assessed using the immunoturbidimetric method. PTH was measured with the Beckman DXI800 analyzer using the chemiluminescent immunoassay method.

For BMI measurement, weight (kg) was divided by the square of height ^2 (m). The current or former smokers within the previous 12 months were identified as having engaged in smoking. Weekly alcohol consumption over the past 12 months was used to identify drinking habits. All patients underwent an 8-hour fasting period before blood collection, during which all experimental measurements were taken.

### Statistics

In the study, categorical data were represented as frequencies (percentages), whereas continuous demographic, laboratory, and clinical information were displayed as means ± SD or medians (25th, 75th). Univariate analyses of categorical data were performed using Pearson’s chi-square tests or Fisher’s exact tests. For continuous data, independent sample t-tests and Mann-Whitney U tests were used, depending on whether the data followed a normal or non-normal distribution. Furthermore, the univariate analysis was performed to explore the correlation between BTMs and the characteristics of OPFs patients hospitalized for surgical intervention.

Appropriate covariate adjustments have been implemented using the generalized estimating equation (GEE) to examine the independent connection between TyG index levels and BTMs in OPFs patients hospitalized for surgical intervention. The models that were developed comprised three variations: Model 1 (unadjusted), Model 2 (minimally adjusted), and Model 3 (completely adjusted). Initially, to identify collinearity among these covariates, a variance inflation factor (VIF) analysis was executed. Afterward, adjustments for these covariates were determined using the following standards: (1) the standardized regression coefficient (β) or matched odds ratio (OR) changed by ≥ 10% when a covariate was included or excluded from the baseline or full model; and (2) if the P-value for the covariate in model 1 or the univariate model was ≤ 0.1.

The identification of possible non-linear correlations was accomplished using a generalized additive model (GAM). In cases where an apparent correlation existed, the threshold effect of the resultant smoothed curve was determined using a two-segment linear regression model. A recursive method was employed to independently compute the inflection point applying a maximum likelihood model in situations where these curves displayed a distinct ratio. Subgroup analyses were also conducted on clinically relevant subsets of study participants to enhance the reliability of the findings. A two-tailed test revealed a significant threshold at P-value ≤ 0.05.

This study utilizes GPT-3.5 as a language refinement tool. A threshold level for all evaluations was noted via a two-sided P-value ≤ 0.05 obtained from the R packages (http://www.R-project.org, The R Foundation) and Empower Stats (http://www.empowerstats.com, X&Y Solutions, Inc, MA, USA).

## Results

### Patient details

The baseline characteristics of 578 OPFs patients hospitalized for surgical intervention between January 1, 2017, and July 27, 2022 (n = 578) are summarized in **Table 1**. The baseline characteristics are presented in the established TyG quartiles. The average age of these patients was 68.94 ± 11.00 years, with 30.80% being male and 69.20% being female. The mean concentrations of P1NP and β-CTX in these patients were measured at 56.26 ± 25.61 ng/mL and 0.53 ± 0.26 ng/mL, respectively. In comparison, the overall patient population had a mean TyG index of 6.96 ± 0.58. The population’s baseline characteristics were analyzed using ascending quartiles of the TyG index (Q1-Q4). There were remarkable variations (*P*-value ≤ 0.05) noted in the levels of TG, FBG, homocysteine, Apo B, P, Ca, UA, LDL, and PTH as the TyG index elevated. In contrast, HDL levels reduced as the TyG index increased.

**Table 1:** Patient characteristics based on TyG index quartiles.

TyG index	Mean±SD / N (%)				*P-*value	*P-*value*
	Q1	Q2	Q3	Q4		
N	145	144	144	145		
Age, years	68.12 ± 10.59	67.69 ± 10.93	69.64 ± 11.00	70.31 ± 11.36	0.138	0.172
BMI, kg/m^2^	23.27 ± 3.46	22.96 ± 3.21	22.72 ± 3.23	22.91 ± 3.38	0.556	0.437
TG, mg/dL	11.80 ± 2.87	17.12 ± 3.11	24.09 ± 5.02	41.56 ± 24.50	<0.001	<0.001
FBG, mg/dL	96.57 ± 15.34	99.97 ± 18.36	105.72 ± 22.45	128.62 ± 52.83	<0.001	<0.001
UA, umol/L	255.41 ± 76.85	263.78 ± 84.85	285.56 ± 86.03	299.80 ± 100.89	<0.001	<0.001
AST, U/L	27.59 ± 24.15	24.49 ± 12.78	22.27 ± 7.41	23.68 ± 8.01	0.018	0.115
Homocysteine, μmol/L	11.93 ± 5.82	13.18 ± 6.04	13.37 ± 5.36	14.24 ± 9.43	0.039	0.014
PTH, ng/L	11.93 ± 5.82	13.18 ± 6.04	13.37 ± 5.36	14.24 ± 9.43	0.039	0.014
Apo B, g/L	0.71 ± 0.17	0.81 ± 0.19	0.91 ± 0.19	0.94 ± 0.21	<0.001	<0.001
Apo A, g/L	1.23 ± 0.23	1.24 ± 0.21	1.23 ± 0.23	1.22 ± 0.21	0.916	0.887
LDL, mmol/L	2.23 ± 0.57	2.54 ± 0.68	2.87 ± 0.65	3.02 ± 0.74	<0.001	<0.001
HDL, mmol/L	1.45 ± 0.31	1.39 ± 0.27	1.30 ± 0.28	1.21 ± 0.25	<0.001	<0.001
P1NP, ng/mL	53.77 ± 26.11	58.17 ± 26.42	57.12 ± 23.89	56.00 ± 25.98	0.504	0.193
β-CTX, ng/mL	0.52 ± 0.28	0.56 ± 0.26	0.56 ± 0.25	0.50 ± 0.24	0.115	0.054
P, mmol/L	1.05 ± 0.17	1.06 ± 0.18	1.09 ± 0.16	1.12 ± 0.21	0.003	0.006
Ca, mmol/L	2.18 ± 0.14	2.21 ± 0.11	2.23 ± 0.11	2.25 ± 0.12	<0.001	<0.001
Mg, mmol/L	0.90 ± 0.09	0.92 ± 0.10	0.92 ± 0.09	0.91 ± 0.09	0.230	0.132
N (%)						
Sex, N (%)					0.081	-
Female	110 (75.86%)	89 (61.81%)	100 (69.44%)	101 (69.66%)		
Male	35 (24.14%)	55 (38.19%)	44 (30.56%)	44 (30.34%)		
Drinking, N (%)					0.035	-
No	140 (96.55%)	133 (92.36%)	140 (97.22%)	143 (98.62%)		
Yes	5 (3.45%)	11 (7.64%)	4 (2.78%)	2 (1.38%)		
Smoking, N (%)					0.129	-
No	135 (93.10%)	132 (91.67%)	141 (97.92%)	135 (93.10%)		
Yes	10 (6.90%)	12 (8.33%)	3 (2.08%)	10 (6.90%)		
ASA, N (%)					0.435	-
1	15 (10.34%)	11 (7.64%)	11 (7.64%)	8 (5.52%)		
2	97 (66.90%)	107 (74.31%)	96 (66.67%)	98 (67.59%)		
≥3	33 (22.76%)	26 (18.06%)	37 (25.69%)	39 (26.90%)		

TyG, index triglyceride-glucose index; SD, standard deviation; Q1, first quartile; Q2, second quartile; Q3, third quartile; Q4, fourth quartile; BMI, body mass index; TG, triglyceride; FBG, fasting blood glucose;UA, uric acid; AST, aspartate aminotransferase; PTH, parathyroid hormone; Apo B, apolipoprotein B; Apo A, apolipoprotein A; LDL, low density lipoprotein; HDL, high density lipoprotein; P1NP, procollagen type I N-terminal propeptide; β-CTX, β-C-terminal telopeptide of type I collagen; P, phosphorus; Ca, calcium; Mg, magnesium; ASA, the score of american society of anesthesiologists.

*P*-value*: Kruskal Wallis Rank Test for continuous variables, Fisher Exact for categorical variables with Expects<10.

### Univariate analyses of factors linked with BTMs

The univariate analyses (**Supplementary Table S1**) revealed a distinct correlation between P1NP and the variables FBG, UA, and P. There were no other variables that showed a correlation with P1NP in OPFs patients hospitalized for surgical intervention. A significant link was also identified in univariate analyses between β-CTX and the following variables: FBG, AST, HDL, UA, P, and BMI ≥ 28. Besides, no other variables exhibited a correlation with BTMs in OPFs patients hospitalized for surgical intervention.

### Exploration of the connection between the TyG index and BTMs

The relationship between the TyG index and P1NP in OPFs patients hospitalized for surgical intervention was further studied using three models (**Table 2**). Based on the findings, it can be observed that the TyG index and P1NP in model 1 do not show a statistically significant correlation (*β* = 0.51, 95% CI: -3.11 to 4.13, *P*-value = 0.783). Moreover, the link between the TyG index and P1NP remained insignificant (*β* = 0.51, 95% CI: -3.13 to 4.14, *P*-value = 0.785) despite controlling for age, gender, and BMI in model 2. Nevertheless, after conducting further modifications to adjust during potential confounding variables, including age, sex, smoking, drinking, BMI, PTH, Apo B, Apo A, HDL, P, Ca, and AST, model 3 identified a statistically significant inverse relationship between TyG index and P1NP (*β* = -4.70, 95% CI: -9.30 to -0.09, P-value = 0.046).

**Table 2:** Association between TyG index and P1NP in different models.

	Adjust 1^a^	Adjust 2^b^	Adjust 3^c^
	β (95% CI) *P-*value	β (95% CI) *P-*value	β (95% CI) *P-*value
TyG index	0.51 (-3.11, 4.13) 0.783	0.51 (-3.13, 4.14) 0.785	-4.70 (-9.30, -0.09) 0.046

a No adjustment.

b Adjusted for age, sex, BMI

c Adjusted for age, sex, BMI, smoking, drinking, PTH; Apo B, Apo A, HDL, P, Ca, AST.

TyG index, triglyceride-glucose index; CI, confidence interval; P1NP, procollagen type I N-terminal propeptide; BMI, body mass index; PTH, parathyroid hormone; Apo B, apolipoprotein B; Apo A, apolipoprotein A; HDL, high density lipoprotein; P, phosphorus; Ca, calcium; AST, aspartate aminotransferase.

The possible relationship between β-CTX and TyG was examined using the defined models, as detailed in **Table 3**. There was no significant relationship between the two variables in model 1 (*β* = -0.02, 95% CI: -0.06 to 0.01, *P*-value = 0.243). The link between the TyG index and β-CTX remained insignificant despite controlling for gender, age, and BMI in model 2 (*β* = -0.02, 95% CI: -0.06 to 0.01, *P*-value = 0.243). Nevertheless, after accounting for other confounding variables, including age, sex, BMI, smoking, drinking, PTH, Apo B, Apo A, HDL, P, Ca, Mg, AST, LDL, homocysteine, ASA, and UA, model 3 listed a significant inverse relation (*β* = -0.06, 95% CI: -0.10 to -0.01, *P*-value = 0.012) between TyG index and β-CTX.

**Table 3:** Association between TyG index and β-CTX in different models.

	Adjust 1^a^	Adjust 2^b^	Adjust 3^c^
	β (95% CI) *P-*value	β (95% CI) *P-*value	β (95% CI) *P-*value
TyG index	-0.02 (-0.06, 0.01) 0.243	-0.02 (-0.06, 0.01) 0.243	-0.06 (-0.10, -0.01) 0.012

a No adjustment

b Adjusted for age, sex, BMI

c Adjusted for age, sex, BMI, smoking, drinking, PTH; Apo B, Apo A, HDL, P, Ca, Mg, AST, LDL, homocysteine, ASA, UA.

TyG, index triglyceride-glucose index; CI, confidence interval; β-CTX, β-C-terminal telopeptide of type I collagen; BMI, body mass index; PTH, parathyroid hormone; Apo B, apolipoprotein B; Apo A, apolipoprotein A; HDL, high density lipoprotein; P, phosphorus; Ca, calcium; Mg, magnesium; AST, aspartate aminotransferase; LDL, low density lipoprotein; ASA, the score of american society of anesthesiologists; UA, uric acid.

### Spline smoothing plot and threshold analyses

**Figure 2:**
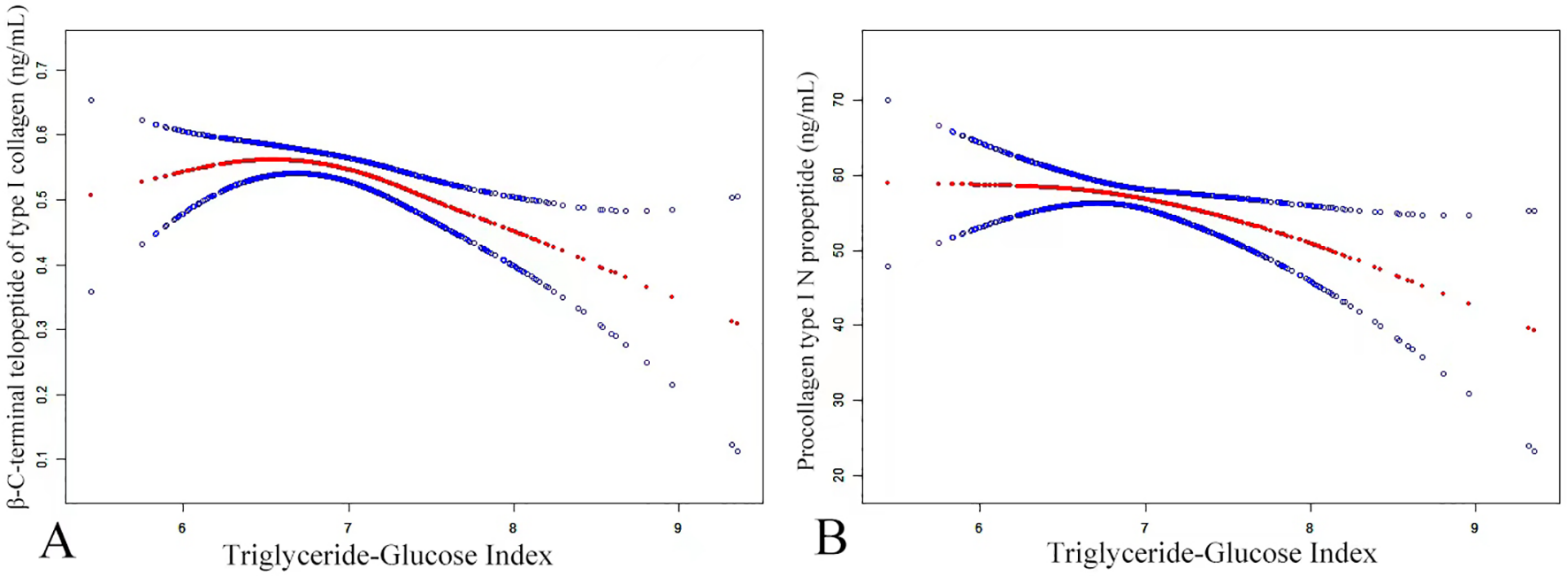
The smoothed adjusted curves for the TyG index, β-CTX **(A)**, and P1NP **(B)**. The red line denotes the non-linear connection between the TyG index and both β-CTX and P1NP, whereas the blue line signifies the CI at 95%. **(A)** A non-linear correlation was identified even after controlling for variables such as age, sex, smoking, drinking, BMI, PTH, Apo B, Apo A, HDL, P, Ca, Mg, AST, LDL, homocysteine, ASA, and UA. **(B)** After controlling for age, sex, smoking, drinking, BMI, PTH, Apo B, Apo A, HDL, P, Ca, and AST, a non-linear connection was noticed.

A threshold effect analysis was performed (**Table 4**) to explore the nature of the relationship between the TyG index and P1NP. After adjusting for potential confounders such as age, sex, BMI, smoking, drinking, PTH, Apo B, Apo A, HDL, P, Ca, and AST, the results revealed a non-linear correlation between the TyG index and P1NP. On the left side of the inflection point (K = 6.63), there was a positive link between the TyG index and P1NP, with an effect size, 95% CI, and *P*-value of 8.45 (-5.48, 22.39) and 0.235, respectively. Conversely, at the inflection point (K = 6.63) on the right side, a negative association was noted between the TyG index and P1NP, with effect size, 95% CI, and P-value of -7.49 (-12.87, -2.11) and 0.007, respectively. (refer to **Figure 2**).

**Table 4:** Threshold analyses examining the relationship between TyG index and Bone turnover markers.

	Model 3^a^	Model 3^b^
	P1NP	β-CTX
	β (95% CI) *P-*value	β (95% CI) *P-*value
Model A^c^		
One line slope	-4.70 (-9.30, -0.09) 0.046	-0.06 (-0.11, -0.02) 0.006
Model B^d^		
TyG index turning point (K)	6.63	6.31
<K	8.45 (-5.48, 22.39) 0.235	0.33 (0.06, 0.59) 0.016
>K	-7.49 (-12.87, -2.11) 0.007	-0.09 (-0.13, -0.04) < 0.001
Slope 2-Slope 1	-15.94 (-31.90, 0.01) 0.051	-0.41 (-0.69, -0.14) 0.004
LRT^e^	0.048	0.003

a Adjusted for age, sex, BMI, smoking, drinking, PTH; Apo B, Apo A, HDL, P, Ca, AST.

b Adjusted for age, sex, BMI, smoking, drinking, PTH; Apo B, Apo A, HDL, P, Ca, Mg, AST, LDL, homocysteine, ASA, UA.

c Linear analysis, *P*-value<0.05 indicates a linear relationship.

d Nonlinear analysis.

e *P*-value<0.05 means Model B is significantly different from Model A, which indicates a nonlinear relationship.

P1NP, procollagen type I N-terminal propeptide; β-CTX, β-C-terminal telopeptide of type I collagen; CI, confidence interval; TyG, index triglyceride-glucose index; BMI, body mass index; PTH, parathyroid hormone; Apo B, apolipoprotein B; Apo A, apolipoprotein A; HDL, high density lipoprotein; P, phosphorus; Ca, calcium; AST, aspartate aminotransferase; Mg, magnesium; LDL, low density lipoprotein; ASA, the score of american society of anesthesiologists; UA, uric acid.

Similarly, a threshold effect analysis was performed to examine the relationship between the TyG index and β-CTX, aiming to determine if it was linear or non-linear. The analysis, adjusted for confounding variables such as age, sex, BMI, smoking, drinking, PTH, Apo B, Apo A, HDL, P, Ca, Mg, AST, LDL, homocysteine, ASA, UA, revealed a non-linear relationship, with an inflection point at K = 6.31. On the left side of this inflection point, a positive association between the TyG index and β-CTX was observed, with an effect size of 0.33 (95% CI: 0.06, 0.59) and a *P*-value of 0.016. Conversely, on the right side of the inflection point, a negative correlation was detected, with an effect size of -0.09 (95% CI: -0.13, -0.04) and a *P*-value of ≤ 0.001.

### Subgroup analysis

To improve the reliability of the findings following the final adjustment in model 3 for potential confounding variables, subgroup analyses were conducted with stratification by age, sex, BMI, smoking, drinking, PTH, Apo B, Apo A, HDL, P, Ca, and AST. Consistent patterns were observed across all strata (**Table 5**), except for the subgroup variables. All analyses were adjusted for the 11 covariates previously listed.

**Table 5:** Subgroup analyses examining the relationship between TyG index and BTMs.

Subgroup	N	P1NP	β-CTX
		β (95% CI) *P-*value	
Age, years			
≤70	329	-0.23 (-5.99, 5.54) 0.938	-0.04 (-0.10, 0.01) 0.130
>70	249	-12.25 (-19.90, -4.60) 0.002	-0.09 (-0.16, -0.01) 0.030
Sex			
Female	400		
Male	178	-4.21 (-13.13, 4.72) 0.357	-0.03 (-0.12, 0.06) 0.535
BMI, kg/m^2^			
<24	348	-7.81 (-13.68, -1.95) 0.009	-0.10 (-0.16, -0.04) <0.001
≥24,≤28	199	-0.70 (-8.48, 7.09) 0.861	-0.01 (-0.09, 0.07) 0.787
>28	31		
Drinking			
No	556	-4.98 (-9.67, -0.30) 0.038	-0.07 (-0.11, -0.02) 0.004
Yes	22		
Smoking			
No	543	-5.03 (-9.81, -0.24) 0.040	-0.07 (-0.12, -0.03) 0.003
Yes	35	11.08 (-6.68, 28.83) 0.234	0.19 (-0.02, 0.41) 0.094
HDL, mmol/L			
≤1.16	168	-6.37 (-13.07, 0.33) 0.064	-0.09 (-0.15, -0.02) 0.015
>1.16,<1.42	206	-5.76 (-14.88, 3.35) 0.217	-0.08 (-0.17, 0.01) 0.074
≥1.42	204	-2.46 (-11.15, 6.23) 0.580	-0.02 (-0.10, 0.06) 0.592
PTH, ng/L			
≤15	423	-3.95 (-9.06, 1.16) 0.130	-0.05 (-0.11, -0.00) 0.039
>15,<65	154	-5.29 (-16.04, 5.46) 0.336	-0.10 (-0.19, -0.00) 0.048
Apo B, g/L			
≤0.8	255	-1.52 (-8.37, 5.34) 0.664	-0.07 (-0.14, 0.00) 0.067
>0.8,<1.1	263	-6.44 (-12.78, -0.10) 0.047	-0.06 (-0.12, -0.00) 0.050
≥1.1	60	-6.52 (-21.93, 8.89) 0.411	-0.01 (-0.16, 0.13) 0.862
Apo A, g/L			
≤1.0	85	-10.60 (-22.07, 0.86) 0.074	-0.09 (-0.24, 0.07) 0.279
>1.0,<1.6	458	-2.75 (-7.77, 2.28) 0.284	-0.06 (-0.10, -0.01) 0.016
≥1.6	35	1.49 (-29.67, 32.65) 0.926	-0.05 (-0.35, 0.24) 0.722
P, mmol/L			
≤0.97	153	0.02 (-6.98, 7.02) 0.996	-0.03 (-0.11, 0.05) 0.528
>0.97,<1.62	423	-6.03 (-11.89, -0.18) 0.044	-0.06 (-0.12, -0.01) 0.030
Ca, mmol/L			
≤2.25	347	-1.90 (-7.94, 4.15) 0.539	-0.03 (-0.09, 0.02) 0.252
>2.25,<2.75	231	-7.83 (-15.15, -0.51) 0.037	-0.10 (-0.17, -0.02) 0.012
AST, U/L			
≤40	538	-5.37 (-10.22, -0.52) 0.030	-0.06 (-0.10, -0.01) 0.021
>40	40		

Adjusted for age, sex, BMI, smoking, drinking, PTH; Apo B, Apo A, HDL, P, Ca, AST except the subgroup variable.

P1NP, procollagen type I N-terminal propeptide; β-CTX, β-C-terminal telopeptide of type I collagen; CI, confidence interval; BMI, body mass index; HDL, high density lipoprotein; PTH, parathyroid hormone; Apo B, apolipoprotein B; Apo A, apolipoprotein A; P, phosphorus; Ca, calcium; AST, aspartate aminotransferase.

## Discussion

OP is particularly common among the elderly, and the aging process contributes to an increase in OP cases (25). Previous research has shown a link between these elevated levels and an increased risk of OP (22). The primary aim of this study was to investigate the relationship between the TyG index and BTMs in OPFs patients hospitalized for surgical intervention. The study also sought to control for potential confounding variables that might affect the observed relationship. The results revealed a significant negative association between the TyG index and both P1NP and β-CTX. Furthermore, a non-linear relationship was identified, with inflection points at 6.63 and 6.31, respectively.

A previous study examined a connection between bone density and TyG index. The increased TyG index has been linked to decreased bone density in diabetic patients (7), non-diabetic individuals in Korea (10), adult Americans (21), healthy Chinese younger aged and non-diabetic individuals (22), and postmenopausal women (23). Even though bone density is the highest priority for OP diagnosis (26). Dual-energy X-ray absorptiometry (DXA) is considered a promising candidate for quantifying BMD (27). The BMD measurement via DXA may be influenced by osteophytes, degenerative alterations in minor joints, and vertebral abnormalities (27). Consequently, BTMs have appeared as a highly beneficial tool for evaluating bone quality and metabolism, and their applications in the detection of OP and fractures are constantly increasing (28, 29). Previous research has also investigated the link between the TyG index and BTMs in patients with type 2 diabetes, revealing a negative relationship between them (7). In the influence of multiple confounding variables, this study evaluated the further connection between the TyG index and BTMs in OPFs patients hospitalized for surgical intervention and found an unfavorable relationship between these two variables. Moreover, a threshold effect and a non-linear connection were observed between the TyG index and BTMs.

Insulin resistance impacts bone cells and disrupts bone formation, likely by inhibiting the Wnt/β-catenin signaling pathway (30). Studies in rodent models have shown that obesity, coupled with insulin resistance, leads to reduced osteoblastic proliferation, increased osteoblastic apoptosis, heightened osteoblastic insulin resistance, and greater bone porosity. These changes result in impaired bone quality, particularly in trabecular bone areas (30).

It has demonstrated that elevated TG and blood glucose levels influence a patient’s bone metabolism in distinct ways, ultimately resulting in reduced bone density and a higher susceptibility to OP. Previous findings have revealed a positive association between triglyceride levels and OP (31), and high TG levels may likely affect bone density via various mechanisms. One possible mechanism involves the activation of peroxisome proliferator-activated receptor γ (PPARγ), a key factor in the relationship between lipid indicators and BMD. The PPARγ is activated by lipid metabolites, and elevated levels of these metabolites can lead to increased PPARγ activity. This, in turn, inhibits bone formation while promoting bone resorption. Another possible mechanism entails the strong correlation between elevated levels of triglycerides in the blood and enhanced fat content in the bone marrow; this correlation may result in a reduction in trabecular bone density (32–34). It can be attributed to the detrimental consequences of oxidative stress, which simultaneously hinder the differentiation of osteoblasts and stimulate the adipocyte differentiation (35, 36).

Hyperglycemia has also been associated with reduced bone density via multiple pathways. Prolonged hyperglycemia in these mechanisms inhibits osteoblast proliferation and stimulates osteoclast differentiation (37). It is currently believed that high glucose levels in the bone marrow microenvironment can lead to an increase in osteoclast differentiation, resulting in the development of osteoporosis (37). Furthermore, elevated blood glucose levels may contribute to a rise in advanced glycation end-products (AGEs), which can bind with the receptor for AGEs (RAGE) on bone cells and react to collagen in the bone. This may result in the generation of oxidative and inflammatory mediators, including nuclear factor κB (NF-κB), which can impair bone biomechanical characteristics and reduce its mass, thereby elevating the susceptibility to fractures. Finally, the differentiation of mesenchymal stem cells (MSCs) and bone development can be influenced by oxidative stress, hyperglycemia (38), and the generation of reactive oxygen species (ROS) (39).

These findings have significant clinical implications. First, a negative connection was identified between BTMs and the TyG index, characterized by a threshold effect and a non-linear relationship. Specifically, markers of bone formation declined when the TyG index exceeded 6.63, while markers of bone resorption decreased when the TyG index surpassed 6.31. These results collectively suggest that elevated TyG index levels have an adverse impact on bone turnover. Therefore, monitoring TyG index levels in patients can provide valuable insights into bone metabolism in OPFs patients hospitalized for surgical intervention. Interventions targeting patients with TyG index levels above these thresholds could potentially improve their bone health and overall quality of life. Furthermore, multiple studies have been carried out to examine the link between the TyG index and BTMs in various populations, including diabetic patients (7), non-diabetic Koreans (10), American adults (21), young-aged non-diabetic Chinese individuals (22), and postmenopausal females (23). Nevertheless, to the best of our understanding, no previous study has examined the link between the TyG index and BTMs in OPFs patients hospitalized for surgical intervention; thus, this study holds considerable therapeutic value in this population. Consistent with previous studies, these findings indicate that an elevated TyG index has an adverse effect on bone turnover.

This study also possesses certain strengths. Initially, three distinct models were established to explore the connection between the TyG index and BTMs with potential confounding variables. The results showed a negative relationship between the TyG index and BTMs in OPFs patients hospitalized for surgical intervention, indicating that a high TyG index level may impair BTMs in this population. Thus, to reduce the high incidence and death rates related to OP and fractures, it may be useful to monitor the TyG index levels of patients for screening and assessing their risk. Moreover, compared to laboratory and imaging tests related to bone density, obtaining the TyG index is simpler, more convenient, and more cost-effective. Nonetheless, the present study has some limitations. To commence, as a result of its cross-sectional design, finding the temporal correlation between the TyG index and the onset of OP unveils a difficult challenge. Secondly, as many genetic and non-genetic factors are linked with the development of OP, the presented data analysis mainly adjusted for certain demographic, lifestyle, and laboratory variables. However, it is important to note that individuals with diseases or those taking medications that affect lipid and glucose metabolism were excluded from the study. As a result, the applicability of these findings to such populations may be limited. Moreover, the sample size was comparatively low, consisting of no more than 578 identifiable samples. Finally, these results may not be applicable to individuals of other races and are only applicable to OPFs patients hospitalized for surgical intervention, as the study was carried out at a single institute on a comparatively small patient population. To ensure the consistency and validity of these findings, it will be necessary to conduct extensive follow-up studies that incorporate multi-center randomized designs, a wider range of biochemical markers, and diverse ethnic populations.

## Conclusion

In conclusion, this study identified a significant correlation and non-linear relationship between BTMs and the TyG index in OPFs patients hospitalized for surgical intervention. The analysis revealed threshold effects at TyG index values of 6.63 and 6.31. These findings suggest that elevated TyG index levels may impair bone turnover and contribute to the development of osteoporosis. However, it is important to acknowledge the limitations of this study, such as the small sample size, lack of mechanistic investigations, and restricted population selection. Therefore, further research is needed to explore the causal relationship between the TyG index and osteoporosis and to validate the accuracy of the TyG index in larger and more diverse patient populations.

## Data Availability

The original contributions presented in the study are included in the article/**Supplementary Material**. Further inquiries can be directed to the corresponding author.
