# Correction: The association between the triglyceride-glucose index and bone turnover markers in osteoporotic fractures patients aged 50 and above who are hospitalized for surgical intervention: a retrospective cross-sectional study

**DOI:** 10.3389/fendo.2026.1894363

**Published:** 2026-06-08

**Authors:** Jian Xu, Shao-han Guo, Min-zhe Xu, Chong Li, Ya-qin Gong, Ke Lu

**Affiliations:** 1 Department of Orthopedics, The First People’s Hospital of Kunshan, Gusu School, Nanjing Medical University, Suzhou, Jiangsu, China; 2 Department of Orthopedics, Affiliated Kunshan Hospital of Jiangsu University, Suzhou, Jiangsu, China; 3 Information Department, Affiliated Kunshan Hospital of Jiangsu University, Suzhou, Jiangsu, China

**Keywords:** P1NP, β-CTX, BTMs, osteoporotic fractures, TyG index

In the published article, affiliation “^2^Department of Orthopedics, Affiliated Kunshan Hospital of Jiangsu University, Suzhou, Jiangsu, China.” was erroneously assigned to author “Ke Lu” instead of the correct affiliation “^1^Department of Orthopedics, The First People’s Hospital of Kunshan, Gusu School, Nanjing Medical University, Suzhou, Jiangsu, China.”

The original version of this article has been updated.

