# Supplementary material for: The association between the triglyceride-glucose index and bone turnover markers in osteoporotic fractures patients aged 50 and above who are hospitalized for surgical intervention: a retrospective cross-sectional study
Source: Front Endocrinol (Lausanne). 2024 Sep 18;15:1418271. doi: 10.3389/fendo.2024.1418271 (PMC11445018; doi:10.3389/fendo.2024.1418271)
Supplement: Supplementary file 1 [file SupplementaryTable1.docx]

**Table S1. Univariate analyses of factors associated with BTMs**

| Characteristics | Statistics | P1NP | β-CTX |
| --- | --- | --- | --- |
|  |  | β (95% CI) *P-*value | |
| Age, years | 68.9 ± 11.0 | 0.0 (-0.2, 0.2) 0.720 | 0.0 (-0.0, 0.0) 0.488 |
| TG,mg/dL | 23.7 ± 16.9 | 0.1 (-0.0, 0.2) 0.165 | -0.0 (-0.0, 0.0) 0.641 |
| FBG,mg/dL | 107.7 ± 33.5 | -0.1 (-0.2, -0.1) <0.001 | -0.0 (-0.0, -0.0) 0.003 |
| UA, umol/L | 276.1 ± 89.1 | -0.0 (-0.1, -0.0) 0.022 | -0.0 (-0.0, -0.0) <0.001 |
| AST,U /L | 24.5 ± 14.8 | 0.1 (-0.0, 0.2) 0.139 | -0.0 (-0.0, -0.0) 0.013 |
| Homocysteine, μmol/L | 13.2 ± 6.9 | 0.2 (-0.1, 0.5) 0.130 | -0.0 (-0.0, 0.0) 0.141 |
| PTH, ng/L | 13.2 ± 6.9 | 0.2 (-0.1, 0.5) 0.130 | -0.0 (-0.0, 0.0) 0.141 |
| Apo B, g/L | 0.8 ± 0.2 | 3.4 (-6.6, 13.3) 0.505 | -0.1 (-0.2, 0.0) 0.297 |
| Apo A, g/L | 1.2 ± 0.2 | 2.7 (-6.8, 12.2) 0.578 | -0.1 (-0.2, 0.0) 0.058 |
| LDL,mmol/L | 2.7 ± 0.7 | -0.4 (-3.3, 2.4) 0.771 | -0.0 (-0.0, 0.0) 0.223 |
| HDL,mmol/L | 1.3 ± 0.3 | -4.0 (-11.2, 3.2) 0.273 | -0.1 (-0.2, -0.0) 0.023 |
| Mg,mmol/L | 0.9 ± 0.1 | 9.4 (-12.7, 31.6) 0.403 | 0.1 (-0.1, 0.3) 0.461 |
| P,mmol/L | 1.1 ± 0.2 | 29.3 (18.1, 40.5) <0.001 | 0.4 (0.3, 0.6) <0.001 |
| Ca,mmol/L | 2.2 ± 0.1 | 8.8 (-8.3, 26.0) 0.313 | 0.1 (-0.1, 0.3) 0.182 |
| TyG index | 7.0 ± 0.6 | 0.5 (-3.1, 4.1) 0.783 | -0.0 (-0.1, 0.0) 0.243 |
| N (%) |  |  |  |
| BMI, kg/m^2^, N (%) |  |  |  |
| ≤24 | 348 (60.2%) | 0 | 0 |
| >24, <28 | 199 (34.4%) | -3.4 (-7.9, 1.0) 0.131 | -0.0 (-0.1, 0.0) 0.158 |
| ≥28 | 31 (5.4%) | 6.3 (-3.0, 15.7) 0.185 | 0.1 (0.0, 0.2) 0.016 |
| Sex, N (%) |  |  |  |
| Female | 400 (69.2%) | 0 | 0 |
| Male | 178 (30.8%) | -0.3 (-4.8, 4.3) 0.905 | 0.0 (-0.0, 0.0) 0.915 |
| Smoking, N (%) |  |  |  |
| No | 543 (93.9%) | 0 | 0 |
| Yes | 35 (6.1%) | -6.4 (-15.1, 2.4) 0.154 | -0.0 (-0.1, 0.1) 0.451 |
| Drinking, N (%) |  |  |  |
| No | 556 (96.2%) | 0 | 0 |
| Yes | 22 (3.8%) | -0.8 (-11.8, 10.1) 0.880 | 0.0 (-0.1, 0.1) 0.962 |
| ASA, N (%) |  |  |  |
| 1 | 45 (7.8%) | 0 | 0 |
| 2 | 398 (68.9%) | 1.1 (-6.8, 9.0) 0.778 | 0.0 (-0.0, 0.1) 0.338 |
| ≥3 | 135 (23.4%) | -0.4 (-9.1, 8.2) 0.924 | 0.0 (-0.1, 0.1) 0.494 |

Abbreviations: P1NP, procollagen type I N-terminal propeptide; β-CTX, β-C-terminal telopeptide of type I collagen; CI, confidence interval; TG, triglyceride; FBG, fasting blood glucose; UA, uric acid; AST, aspartate aminotransferase; PTH, parathyroid hormone; Apo B, apolipoprotein B; Apo A, apolipoprotein A; LDL, low density lipoprotein; HDL, high density lipoprotein; Mg, magnesium; P, phosphorus; Ca, calcium; TyG index, triglyceride-glucose index; BMI, body mass index; ASA, the score of american society of anesthesiologists.
