# Supplementary material for: The association between the triglyceride-glucose index and bone turnover markers in osteoporotic fractures patients aged 50 and above who are hospitalized for surgical intervention: a retrospective cross-sectional study
Source: Front Endocrinol (Lausanne). 2024 Sep 18;15:1418271. doi: 10.3389/fendo.2024.1418271 (PMC11445018; doi:10.3389/fendo.2024.1418271)
Supplement: Supplementary file 2 [file SupplementaryTable2.docx]

**Table S2. Association between TyG index and BTMs in different models after adding vitamin D**

**as a covariate**

| P1NP | Adjust 1^a^ | Adjust 2^b^ | Adjust 3^c^ |
| --- | --- | --- | --- |
|  | β (95% CI) *P-*value | β (95% CI) *P-*value | β (95% CI) *P-*value |
| TyG index | 0.51 (-3.11, 4.13) 0.783 | 0.51 (-3.13, 4.14) 0.785 | -4.61 (-11.43, 2.21) 0.186 |

^a^No adjustment

^b^Adjusted for age, sex, BMI

^c^Adjusted for age, sex, BMI, smoking, drinking, PTH; Apo B, Apo A, HDL, P, Ca, AST, 25 (OH) D.

| β-CTX | Adjust 1^a^ | Adjust 2^b^ | Adjust 3^c^ |
| --- | --- | --- | --- |
|  | β (95% CI) *P-*value | β (95% CI) *P-*value | β (95% CI) *P-*value |
| TyG index | -0.02 (-0.06, 0.01) 0.243 | -0.02 (-0.06, 0.01) 0.243 | -0.04 (-0.11, 0.03) 0.305 |

^a^No adjustment

^b^Adjusted for age, sex, BMI

^c^Adjusted for age, sex, BMI, smoking, drinking, PTH; Apo B, Apo A, HDL, P, Ca, Mg, AST, LDL, homocysteine, ASA, UA, 25 (OH) D.

Abbreviations: TyG index, triglyceride-glucose index; CI, confidence interval; P1NP, procollagen type I N-terminal propeptide; BMI, body mass index; PTH, parathyroid hormone; Apo B, apolipoprotein B; Apo A, apolipoprotein A; HDL, high density lipoprotein; P, phosphorus; Ca, calcium; AST, aspartate aminotransferase; 25(OH)D, 25-hydroxyvitamin D; Mg, magnesium; LDL, low density lipoprotein; ASA, the score of american society of anesthesiologists; UA, uric acid.
